# Asian-Pacific consensus on small intestinal bacterial overgrowth in gastrointestinal disorders: An initiative of the Indian Neurogastroenterology and Motility Association

**DOI:** 10.1007/s12664-022-01292-x

**Published:** 2022-10-10

**Authors:** Uday C. Ghoshal, Sanjeev Sachdeva, Ujjala Ghoshal, Asha Misra, Amarender Singh Puri, Nitesh Pratap, Ayesha Shah, M. Masudur Rahman, Kok Ann Gwee, Victoria P Y Tan, Tahmeed Ahmed, Yeong Yeh Lee, B S Ramakrishna, Rupjyoti Talukdar, S V Rana, Saroj K Sinha, Minhu Chen, Nayoung Kim, Gerald Holtmann

**Affiliations:** 1 Department of Gastroenterology, Sanjay Gandhi Postgraduate Institute of Medical Sciences, Lucknow, 226 014 India; 2 Department of Gastroenterology, G B Pant Hospital, New Delhi, 110 002 India; 3 Department of Microbiology, Sanjay Gandhi Postgraduate Institute of Medical Sciences, Lucknow, 226 014 India; 4 KIMS Hospital, Secunderabad, 500 003 India; 5 University of Queensland, Faculty of Medicine, and Princess Alexandra Hospital, Department of Gastroenterology and Hepatology, Brisbane, Queensland, Australia; 6 Sheikh Russel National Gastroliver Institute and Hospital, Dhaka, Bangladesh; 7 Department of Medicine, Yong Loo Lin School of Medicine, National University of Singapore, Singapore, Singapore; 8 Stomach, Liver and Bowel Centre, Gleneagles Hospital, Singapore, Singapore; 9 Faculty of Medicine, University of Hong Kong, Pok Fu Lam, Hong Kong; 10 International Centre for Diarrhoeal Disease Research, Bangladesh, Dhaka, Bangladesh; 11 School of Medical Sciences, Universiti Sains Malaysia, Kota Bharu, Malaysia; 12 GI Function and Motility Unit, Hospital Universiti Sains Malaysia, Kota Bharu, Malaysia; 13 SIMS Institute of Gastroenterology, Hepatology, and Transplantation, SRM Institutes for Medical Science, Chennai, 600 026 India; 14 Department of Gastroenterology, Asian Institute of Gastroenterology, Hyderabad, 500 082 India; 15 Department of Biochemistry, All India Institute of Medical Sciences, Rishikesh, 249 203 India; 16 Department of Gastroenterology, Postgraduate Institute of Medical Education and Research, Chandigarh, 160 012 India; 17 Department of Gastroenterology and Hepatology, The First Affiliated Hospital of Sun Yat-sen University, Guangzhou, China; 18 Department of Internal Medicine, Seoul National University Bundang Hospital, Seoul National University College of Medicine, Seoul, South Korea

**Keywords:** Breath methane, Disorders of gut-brain interaction, Dysbiosis, FODMAP, Gut microbiota, Hydrogen breath test, Irritable bowel syndrome, Rifaximin

## Abstract

In the clinical setting, small intestinal bacterial overgrowth (SIBO) is a frequent, but under-diagnosed entity. SIBO is linked to various gastrointestinal (GI) and non-GI disorders with potentially significant morbidity. The optimal management of SIBO is undefined while there is a lack of published consensus guidelines. Against this background, under the auspices of the Indian Neurogastroenterology and Motility Association (INMA), formerly known as the Indian Motility and Functional Diseases Association (IMFDA), experts from the Asian-Pacific region with extensive research and clinical experience in the field of gut dysbiosis including SIBO developed this evidence-based practice guideline for the management of SIBO utilizing a modified Delphi process based upon 37 consensus statements, involving an electronic voting process as well as face-to-face meetings and review of relevant supporting literature. These statements include 6 statements on definition and epidemiology; 11 on etiopathogenesis and pathophysiology; 5 on clinical manifestations, differential diagnosis, and predictors; and 15 on investigations and treatment. When the proportion of those who voted either to accept completely or with minor reservations was 80% or higher, the statement was regarded as accepted. The members of the consensus team consider that this guideline would be valuable to inform clinical practice, teaching, and research on SIBO in the Asian-Pacific region as well as in other countries.

## Introduction

The human gut is inhabited by an intricate population of microbes, collectively known as microbiota. The composition of microbiota in the proximal gut differs qualitatively as well as quantitatively from that in the colon [1]. Usually, the small intestine is devoid of coliform bacteria, and even if present the number is little. Small intestinal bacterial overgrowth (SIBO) is characterized by the presence of an excessive amount of bacteria within the small intestine, which may result in a constellation of gastrointestinal (GI) symptoms [2, 3].

Studies utilizing molecular techniques suggest that compared to the true prevalence, SIBO remains in the clinical setting frequently undiagnosed [4]. Etiopathogenesis of SIBO is multifactorial [1] and it is linked to several GI and non-GI disorders with significant morbidity including irritable bowel syndrome (IBS), non-alcoholic fatty liver disease (NAFLD), chronic pancreatitis, celiac disease, obesity, and inflammatory bowel disease (IBD) [5]. Contrasting the prevalence, research into SIBO has just started and this condition remains worldwide largely under-researched.

While diagnostic modalities for SIBO are still evolving, its management also remains a challenge with the limited data and the absence of consensus-based clinical guidelines. Against this background and under the auspices of the Indian Neurogastroenterology and Motility Association (INMA), formerly known as the Indian Motility and Functional Diseases Association (IMFDA), experts from the Asian-Pacific Region with clinical and research experience in the field of SIBO and gut dysbiosis collaborated with the aim to develop evidence-based practice guideline for the management of patients with SIBO. The consensus team aimed to provide valid guidance for clinical practice, teaching, and future research on SIBO across the globe with the first consensus-based guideline that utilized a rigorous Delphi process.

## Methods

The members of the consensus team were selected from Asian-Pacific countries based on their interest and experience in the field of gut dysbiosis including SIBO as evidenced by an electronic literature search on PubMed. The members included experts from India, Bangladesh, China, South Korea, Singapore, Hong Kong, Malaysia, and Australia. A core group of four members was selected from among the consensus team members who made the first set of 37 statements on definition, epidemiology, etiopathogenesis, pathophysiology, clinical manifestations, differential diagnosis, predictors, investigations, and treatment. The members of the core team had a preliminary face-to-face meeting in Lucknow (India) on 10th November 2017 to develop preliminary statements for further refinement and discussion by the full consensus group.

The consensus process involved a modified Delphi method [6]. Before the first round of voting on the statements, an electronic library was created in the Digital Medical Education section of the Shanti Public Educational and Development Society website (www.spreadhealth.in). The first round of online voting was held in July 2019. The voting was conducted in an electronic online anonymous voting system developed in the Research and Innovation initiative menu in the www.spreadhealth.in and the results were analyzed electronically. The result of the first round of voting was presented to the entire consensus team in a face-to-face meeting held in Kolkata (India) on 13th December 2019, on the sidelines of the Asian-Pacific Digestive Week-2019 (APDW-2019) Conference. During this face-to-face meeting at Kolkata, a discussion was held on the modification of five statements, which could not reach 80% acceptance during the first round of voting. Also, the formulation of an algorithm for the management of SIBO was discussed. The second round of online voting was subsequently held in January 2020 in which the five modified statements were put to voting. Method of Grading of Recommendations, Assessment, Development and Evaluation (GRADE) Working group was used for deriving at the level of agreement, level of evidence, and grade of recommendation (Table 1) [7]. When the proportion of those who voted either to accept completely or with some reservation was 80% or higher, the statement was regarded as accepted. Finally, a consensus was achieved on all 37 statements, which included the five modified statements. An algorithm of management of SIBO was finalized as per suggestions of the consensus team. However, due to the pandemic of Corona Virus Disease-19 (COVID-19) that devastated the whole world, further works on this consensus got stalled for the next 2 years. On 7th May 2022, the core group members of the consensus team physically met during the 5th Annual Congress of the Indian Motility and Functional Diseases Association (now named as INMA) in Lucknow, India, to finalize the manuscript for publication. The consensus was presented on the same day to all the delegates of the 5th INMA Congress.

**Table 1:** Level of the agreement, level of evidence, and grade of recommendation used in this consensus (method of Grading of Recommendations, Assessment, Development and Evaluation [GRADE] working group)

Level of agreement	
I	Accepted completely
II	Accepted with some reservation
III	Accepted with major reservation
IV	Rejected with reservation
V	Rejected completely
Level of evidence	
I	Evidence obtained from at least one randomized controlled trial
II-1	Evidence obtained from well-designed controlled trials without randomization
II-2	Evidence obtained from well-designed cohort or case-controlled study
II-3	Evidence obtained from the comparison between time and places with or without intervention
III	The opinion of respected authorities, based on experience or expert committees
Recommendation (based on the quality of evidence)	
A	There is good evidence to support the statement
B	There is fair evidence to support the statement
C	There is poor evidence to support the statement but recommendation made on other grounds
D	There is fair evidence to refute the statement
E	There is good evidence to refute the statement

## Consensus statements

### Definition and epidemiology

**Statement 1**: Small intestinal bacterial overgrowth (SIBO) is defined as the growth of bacteria ≥ 10^5^ colony-forming unit (CFU)/mL or ≥ 10^3^ CFU/mL (particularly if coliforms are present) on a quantitative culture of upper gut aspirate.

**Voting summary:** Accepted completely 57.9%, accepted with some reservation 36.8%, accepted with major reservation 5.3%.

**Level of evidence:** II-2.

**Grade of recommendation**: B.

SIBO is a clinical condition caused by the presence of an excessive amount of bacteria within the small intestine. It is defined as the growth of bacteria ≥10^5^ CFU/mL or ≥10^3^ CFU/mL (particularly if coliforms are present) on a quantitative culture of upper gut aspirate [1, 8–14]. Healthy controls have generally <10^3^CFU/mL in the upper bowel aspirate culture. However, all these cut-offs need large, good-quality validation studies from different populations across the globe.

Traditionally, a cut-off of ≥10^5^ CFU/mL has been used to define SIBO, but some authors find it too rigorous for conditions other than blind loop syndrome [10]. Hence, a few investigators have tested lower thresholds (≥10^3^ CFU/mL) in studies of SIBO in varied disorders [13–17]. Researchers from North America have recently suggested a lower cut-off of ≥10^3^ CFU/mL for diagnosis of SIBO [18, 19]. The cut-off of ≥10^3^ CFU/mL proposed by this group may be more relevant, especially when performing culture of duodenal aspirate as bacterial counts in the duodenum are expected to be lower due to more acidic environment than that in the jejunum [2]. A recent 16S ribosomal RNA (rRNA) gene sequencing–based study found that culture-based cut-off of >10^3^CFU/mL for SIBO correlated well with clinical symptoms, breath test results, and sequencing [20].

**Statement 2:** SIBO can be high- or low-threshold depending upon the bacterial counts.

**Voting summary:** Accepted completely 100%.

**Level of evidence:** III.

**Grade of recommendation**: C.

The culture-based threshold for SIBO has been contentious, both as per published data as well as the opinion of the concerned experts. Several investigators have put forward their viewpoint of the low- or high-threshold for SIBO based upon the bacterial counts in the small bowel aspirate [13, 15–17]. Low threshold is mostly taken as counts ≥10^3^ CFU/mL, while a high-threshold implies counts ≥10^5^ CFU/mL. The use of varying thresholds has yielded different rates of prevalence of SIBO in several studies. It is quite understandable that the use of a lower threshold often results in a higher frequency of SIBO in the clinical studies reported to date.

A study from Sweden reported a SIBO prevalence of 4% in both IBS patients and controls while using the conventional threshold of ≥10^5^ CFU/mL, but the prevalence was found to be significantly different (43% in IBS vs. 12% in controls) when the lower threshold was used [15]. In a vital study from India on 80 subjects with IBS, 15/80 (19%) had SIBO as per the conventional threshold of ≥10^5^ CFU/mL, while 19/80 (23.8%) additional patients had low-grade or low-threshold SIBO (bacterial counts of ≥10^3^ to 10^5^ CFU/mL) [13]. In a study from the USA on 139 patients with unexplained gas, bloating and diarrhea, the prevalence of SIBO using low- and high-thresholds was 44.6% and 18%, respectively [16]. In a study from India in patients with non-alcoholic steatohepatitis (NASH), jejunal aspirate culture on 35 subjects yielded SIBO in 14/35 (40%) when a low-threshold was used, but the prevalence was only 5/35 (14.3%) when the high-threshold was used [17]. A recent North American consensus on breath testing and American College of Gastroenterology guideline document on SIBO proposed using a lower threshold, i.e. ≥10^3^ CFU/mL in upper gut aspirate culture for diagnosis of SIBO [18, 19].

**Statement 3:** Microbiological spectrum in SIBO may vary based on the underlying causes.

**Voting summary:** Accepted completely 68.3%, accepted with some reservation 21.1%, accepted with major reservation 5.3%, rejected with reservation 5.3%.

**Level of evidence:** II-2.

**Grade of recommendation**: B.

Based on the type of microflora, the cultured bacteria in subjects with SIBO can be broadly classified as Gram-positive flora and coliform flora [21, 22]. Isolated Gram-positive flora may include Streptococcus, Staphylococcus, Enterococcus, Micrococcus, Lactobacillus, Corynebacterium, Fusobacterium, and Peptostreptococcus, while predominant among Gram-negative flora are *Escherichia coli (E. coli)*, Klebsiella, Proteus, Acinetobacter, Enterobacter, Citrobacter, Neisseria, Bacteroides, and Clostridia. There may be a mix of Gram-positive and Gram-negative populations, as well as aerobic and anaerobic bacteria [16, 23].

The type of isolated bacterial species may vary depending upon the underlying pathophysiology [21, 24–26]. While depletion of the gastric acid barrier due to hypochlorhydria, use of proton pump inhibitors (PPIs), or other causes may predispose to SIBO with Gram-positive flora [24, 25], pathophysiological mechanisms like small bowel anatomical alterations and sub-optimal intestinal clearance function predisposes to SIBO with predominantly Gram-negative flora [26].

In a study from Norway [24] on fifteen healthy subjects with a mean age of 84 years, 12 (80%) were found to have hypochlorhydria with mean pH of 6.6 and a mean bacterial count of 10^8^ CFU/mL in fasting gastric aspirate. Normochlorhydric individuals had counts of ≤10^1^ CFU/mL. Predominant microflora detected included *Streptococcus viridans*, coagulase-negative staphylococci, and Haemophilus species. *E. coli* and Klebsiella were found in only one individual. No subject had strict anaerobes in culture of aspirate.

Similarly, data from Switzerland [25], utilizing duodenal aspirate culture performed on 25 patients with peptic ulcer disease taking omeprazole for more than 5 weeks, and 15 control subjects who were outpatients referred for upper GI endoscopy but with no exposure to PPIs found SIBO in 56% of subjects on PPI and 0% of controls. Hemolytic and non-hemolytic streptococci were the most commonly isolated bacteria.

Various etiologies of malabsorption syndrome (MAS) are associated with intestinal stasis that may result in SIBO [27, 28]. In a study from Lucknow (India) [26], jejunal aspirate cultures of 50 patients with MAS were analyzed. The culture showed growth of bacteria in 34/50 (68%) subjects with MAS with 21/50 (42%) having counts ≥10^5^ CFU/mL. The commonest isolated bacteria were Streptococcus species and *E. coli*.

**Statement 4:** The frequency of SIBO is low among healthy subjects but is higher in the elderly.

**Voting summary:** Accepted completely 84.1%, accepted with some reservation 5.3%, accepted with major reservation 5.3%, rejected with reservation 5.3%.

**Level of evidence:** II-2.

**Grade of recommendation**: B.

As per different published studies with an evaluation of small sets of healthy subjects as controls, SIBO has been reported in 0% to 22% depending primarily on the type of diagnostic test used [29, 30]. Frequency of SIBO is higher in the elderly ranging from 14.5% to 56% [31, 32]. A cross-sectional survey from Germany revealed SIBO in 15.6% in older adults, compared with 5.9% in subjects aged 24 to 59 years [33]. Several studies on SIBO in GI disorders have found older age to be an independent risk factor for the occurrence of SIBO [34–36].

Elderly subjects are expected to be more prone to SIBO because of several factors like reduced gastric acid [24], reduced GI motility, anatomic factors like diverticula, co-morbidities like diabetes mellitus, and use of various medications, which may predispose to SIBO. A study from the UK [37] found that factors predictive of a positive glucose hydrogen breath test (GHBT) in the elderly included increasing age (>75 years), low serum vitamin B_12_, low serum albumin, previous partial gastrectomy, previous right hemicolectomy, presence of small bowel diverticula, and concurrent use of a PPI. In fact, a study from Lucknow, India, showed that lower levels of hemoglobin were associated with a greater likelihood of SIBO [38], which is commensurate with the data on the elderly showing low serum B_12_ to be predictive of the presence of SIBO.

**Statement 5:** In several conditions, the prevalence of SIBO is higher than in healthy controls.

**Voting summary:** Accepted completely 89.4%, accepted with some reservation 5.3%, accepted with major reservation 5.3%.

**Level of evidence:** II-2.

**Grade of recommendation**: A.

The true prevalence of SIBO in the general population or even in at-risk groups is not precisely established [29, 39]. Overall, as a clinical entity, it is still under-recognized. It has been associated with a plethora of gastroenterological as well as non-gastroenterological disorders [2]. The most prominent GI conditions linked with SIBO include IBS, IBD, tropical sprue, celiac disease, dyspepsia, small bowel diverticulosis/stricture/fistula, radiation enteropathy, pancreatitis, NAFLD, liver cirrhosis, and post-abdominal surgery. The most eminent non-GI disorders reported to be associated with SIBO include systemic sclerosis, diabetes mellitus, hypothyroidism, obesity, Parkinson’s disease, multiple sclerosis, muscular dystrophy, end-stage renal failure, coronary artery disease, immunodeficiency syndromes, chronic fatigue syndrome, restless leg syndrome, fibromyalgia, and rosacea [30, 39]. ^.^ More recently, utilizing a culture-independent technique, some of the authors found higher bacterial load in patients with functional GI disorders as compared to controls [4].

The frequency of SIBO in these associated disorders is highly variable, ranging from as low as 4% to as high as 93% [2, 30]. Many of these studies, which included a small set of healthy subjects as controls, reported SIBO in 0% to 22% in such subjects [29, 30]. A few more interesting clinical entities have been recently added to the list of associations with SIBO. These include environmental enteropathy, familial Mediterranean fever, deep venous thrombosis, *Helicobacter pylori* infection, gallstone disease, post-cholecystectomy, and post-colectomy states [39–46]. A recent study from France reported a very high (83%) prevalence of SIBO in a cohort of patients with abdominal symptoms following Roux-en-Y gastric bypass for obesity [47].

**Statement 6:** Among specific disorders associated with SIBO, the reported prevalence differs substantially based on the population studied and the diagnostic method/criteria used to diagnose SIBO.

**Voting summary:** Accepted completely 94.7%, accepted with some reservation 5.3%.

**Level of evidence:** II-2.

**Grade of recommendation**: A.

In particular, disorders associated with SIBO, the published prevalence differs considerably based on the study population and type of diagnostic modality [11, 29, 48]. Such large variability in the prevalence of SIBO in disease populations as well as in healthy controls is primarily due to limitations of current diagnostic technologies [39]. If the breath test is used for evaluation, the frequency of SIBO varies further depending on the nature and dosage of the test substrate used for testing. If upper gut aspirate culture analysis is used for diagnosis, then the diagnostic threshold used for diagnosis often decides the prevalence rates. A recent meta-analysis [49] which included 19 case-control studies of SIBO in chronic liver disease (CLD) found the prevalence of SIBO in CLD using the breath tests was 35.80% compared with 8.0% in controls. However, with culture-based methods, the prevalence was 68.31% in CLD patients as compared with 7.94% in controls.

The best GI disorder which exemplifies the essence of this statement is IBS. Posserud et al. reported a SIBO prevalence of only 4% in IBS patients and controls while using the culture threshold of ≥10^5^ CFU/mL, but the prevalence was 43% in IBS vs. 12% in controls when a lower threshold of ≥10^3^ CFU/mL was used [15]. Ghoshal et al., in a study on 80 patients with IBS, found SIBO in 15/80 (19%) as per the conventional threshold of ≥10^5^ CFU/mL in upper gut aspirate culture [13, 14]. Assuming culture to be the gold standard, 4/15 (27%) with and none of 65 without SIBO had a positive result on GHBT (sensitivity 27%, specificity 100%). None of 15 with and one of 65 without SIBO had documented double peaks on lactulose hydrogen breath test (LHBT) (sensitivity 0%, specificity 98%); and 5/15 (33%) with and 23/65 (35%) without SIBO had an early peak documented on LHBT (sensitivity 33%, specificity 65%) [13]. A very recent systematic review and meta-analysis summarizing 6 case-control and cohort studies also pointed towards a link between SIBO and functional dyspepsia [50].

In a meta-analysis of 12 studies of SIBO in IBS [51], with 1921 subjects, the pooled prevalence of a positive LHBT or GHBT was 54% and 31%, respectively while the prevalence of a positive jejunal aspirate and culture was only 4%. The prevalence in IBS compared with the control subjects varied according to the criteria used to define a positive test. A recent review reported wide variations in prevalence of SIBO as per culture/GHBT/LHBT in controls (0–4%/1–13%/7–40%) and patients with IBS (4.3–44.6%/6.2–45.8%/10–78%) [48]. A recent meta-analysis of fifty studies of SIBO in IBS showed that pooled prevalence of SIBO in IBS was higher in studies diagnosed by breath tests (40%) compared with aspirate cultures (19%) [36]. Another meta-analysis [52] that included 25 studies found that SIBO prevalence in patients with IBS vs. controls showed wide variability depending on the diagnostic method used (breath testing: 35.5% vs. 29.7%; culture with ≥10^5^ CFU/mL cut-off: 13.9% vs. 5.0%; culture with ≥10^3^ CFU/mL cut-off: 33.5% vs. 8.2%). SIBO prevalence diagnosed by LHBT was much higher in both IBS (3.6-folds) and controls (7.6-folds) compared to GHBT. Similarly, LHBT over-diagnosed SIBO as compared to culture. The most recent meta-analysis [53] analyzed 37 studies and found an overall prevalence of SIBO in IBS as 36.7% (range: 4.3% to 83.7%). The range of prevalence as per GHBT, LHBT, and upper gut aspirate culture was 6.2% to 45.8%, 18.4% to 83.7%, and 4.3% to 44.6%, respectively. In addition, IBD also appears to be associated with SIBO [54], which has also been shown in a recent study from India [55]. Patients with Crohn’s disease more often had SIBO than those with ulcerative colitis in this study [55].

### Etiopathogenesis and pathophysiology

**Statement 7:** Disruption of endogenous gut defense mechanisms is known to result in SIBO.

**Voting summary:** Accepted completely 52.6%, accepted with some reservation 36.8%, accepted with major reservation 10.6%.

**Level of evidence:** II-2.

**Grade of recommendation**: B.

Several host defense mechanisms help to prevent SIBO. These include gastric acid, bacteriostatic properties of pancreatobiliary secretions, maintained GI motility especially the coordinated antegrade small bowel peristalsis with intact migratory motor complex (MMC), intact structure and function of the ileocecal cecal valve, salivary immunoglobulin (Ig)-A, pentavalent IgA in the intestinal secretions, defensins from Paneth cells, protective intestinal mucus layer, intact intestinal mucosal immune system, and protective effects of some beneficial commensal flora like lactobacilli [1, 11, 19, 21, 29]. Disruption or deficiency of any one or more of these protective mechanisms may result in the development of SIBO.

Loss of protective gastric acid barrier for any reason may result in SIBO, especially with Gram-positive bacteria [21, 24]. In vitro and in vivo studies indicate that more than 99% of the ingested bacteria get killed within minutes by the physiological gastric acid barrier thus, preventing their colonization in the upper GI tract [56]. Hypochlorhydia or achlorhydria due to any cause like aging, autoimmune gastritis, *H. pylori*–related atrophic gastritis, or partial or total gastrectomy increases the risk of SIBO [19, 24, 57–59]. In a study from Norway [24] on fifteen healthy elderly subjects, 12 (80%) were found to have hypochlorhydria and had an average bacterial count of 10^8^ CFU/mL in fasting gastric aspirate as against counts of ≤10^1^ CFU/mL in normochlorhydric subjects. Predominant bacteria detected were Gram-positive. A case-control study from Korea [58] compared the prevalence of SIBO in post-gastrectomy patients and controls using GHBT. The frequency of SIBO was significantly higher in these patients than in controls (77.6% vs. 6.7%). SIBO was shown to be associated with postprandial intestinal symptoms and late hypoglycemia. Hypochlorhydria caused by treatment with PPI has been reported to be associated with SIBO in several studies [25, 60–64]. However, a few other studies had different conclusions [65–67].

Small bowel motility with intact antegrade peristalsis is a vital protective mechanism against SIBO. Any primary or acquired disorder that hampers the enteric neuromuscular system may result in bacterial overgrowth [21]. Disorders like scleroderma, diabetes mellitus, hypothyroidism, and drugs like opioids may disrupt this critical defense mechanism, thus predisposing to SIBO [35, 68–77]. Prolongation of oro-cecal transit time (OCTT) and small bowel transit time (SBTT) have both been shown to be associated with SIBO [73–75, 78–82]. Intact competence of ileocecal valve has also been shown to be an important defense against SIBO [82, 83].

Since intestinal mucosal immunity regulates gut microbiota, its disruption may predispose to SIBO. But there are limited studies to support this issue. Local (GI) and systemic immune deficiency have both been reported to be associated with an increased risk of SIBO [84–86]. A study from Italy evaluated SIBO in children with IgA deficiency, T-cell deficiency, and hypogammaglobulinemia [86]. SIBO was detected in 5/12 (41.6%) using an upper gut aspirate culture [86]. A study from Mexico evaluated 18 adult patients with small bowel nodular lymphoid hyperplasia [87]. Nine patients (50%) had evidence of immunodeficiency and SIBO was detected in 3 (17%) subjects.

**Statement 8:** Several disorders that alter GI motility may lead to SIBO.

**Voting summary:** Accepted completely 89.4%, accepted with some reservation 5.3%, accepted with major reservation 5.3%.

**Level of evidence:** II-1.

**Grade of recommendation**: A.

Normal small intestinal motility driven by MMC and coordinated antegrade peristalsis helps in the propulsion of food, microbes, and secretions, thus protecting against bacterial or fungal overgrowth [19, 88]. Slowing of intestinal motility either by drugs or disease conditions leads to stagnation of intraluminal contents, which assists in the development of SIBO [70, 81]. Origin of intestinal dysmotility may be neuropathic or myopathic, which in turn can be either primary or secondary to a variety of disorders like scleroderma, amyloidosis, muscular dystrophy, radiation enteropathy, and even paraneoplastic syndrome [21, 89]. Chronic intestinal pseudo-obstruction (CIPO) represents a most severe form of many of these underlying disorders. Several other systemic disorders like diabetes mellitus, hypothyroidism, chronic renal failure, and neurodegenerative conditions (Parkinsonism, multiple sclerosis) can affect GI motility and predispose to SIBO [11, 39]. Dysfunction of the ileocecal valve has also been shown to contribute to the pathogenesis of SIBO [82]. An interesting study from the USA [63] evaluated subjects with unexplained GI symptoms and found GI dysmotility and PPI use to be independent predictors of SIBO or small intestinal fungal overgrowth (SIFO).

The prototype of disorders that predispose to SIBO by altering GI motility is systemic sclerosis (SSc). A study from Italy evaluated 55 patients with SSc and 60 healthy controls [68]. LHBT was used for the assessment of OCTT and SIBO. Frequency of SIBO was significantly higher in SSc than in controls (55.6% vs. 6.7%) and average OCTT was significantly longer in SSc than in controls (150 vs. 105 min) suggesting impaired intestinal motility in SSc. A study from France [69] evaluated 51 subjects with SSc for SIBO using GHBT. The prevalence of SIBO was 43.1% and predictors of SIBO included the presence of diarrhea or constipation. Another study from France used GHBT to evaluate SIBO in 37 subjects with SSc having GI symptoms [70]. Prevalence of SIBO in these subjects was found to be 38%, and predictors of SIBO included longer disease duration and significant weight loss in the last 6 months. A recent study from Thailand evaluated 89 patients with SSc for SIBO using GHBT [90]. Of 89 patients included in this study, 12 had SIBO by GHBT yielding prevalence of 13.5% and longer disease duration was a predictor of SIBO [90]. A study from Chandigarh (India) performed GHBT for SIBO and LHBT for OCTT in 37 patients with SSc [91]. SIBO was found in 7/37 and OCTT was prolonged in 23/37 patients. A recent systematic review from Poland analyzed 7 studies on this issue [71]. The analysis showed a pooled prevalence of SIBO of 39% in patients suffering from SSc. Longer duration of illness predisposed to SIBO in these patients.

Another important systemic condition that may predispose to SIBO by affecting GI motility is diabetes mellitus. A case-control study from Chandigarh (India) evaluated the frequency of SIBO in 84 patients with diabetes mellitus and 45 controls using GHBT) [73]. In addition, an estimate was made of the OCTT. The prevalence of SIBO in patients was significantly higher than in controls (15.5% vs. 2.2%). OCTT was significantly higher in diabetic patients with SIBO. In another case-control study from India, 175 patients with diabetes mellitus and an equal number of healthy controls were evaluated for SIBO and OCTT using GHBT and LHBT, respectively [74]. The prevalence of SIBO was significantly higher in patients as compared to controls (14.8% vs. 2.8%). The OCTT in diabetic patients was significantly longer than in controls, and that in patients with SIBO was higher than in those without SIBO. In another case-control study, Malik et al. evaluated 75 patients with type I diabetes mellitus and 75 healthy subjects using GHBT (for SIBO) and LHBT (for OCTT) [75]. They also measured plasma levels of inflammatory cytokines and anti-oxidants, as well as oxidative stress parameters, to assess the interplay of SIBO with these factors. The prevalence of SIBO was significantly higher in patients as compared to controls (22.7% vs. 1.3%). The OCTT in patients was significantly higher than in controls, and that in patients with SIBO was higher than those without SIBO. Levels of inflammatory cytokines, superoxide dismutase, and catalase were significantly higher but levels of reduced glutathione significantly lower in patients as compared to controls. A study from Italy [72] evaluated 74 subjects with diabetes using LHBT and found that 21 of them had SIBO and delayed OCTT. These 21 subjects were tested again with LHBT after a course of rifaximin. Sixty-two percent of patients showed a significant decrease in OCTT along with eradication of SIBO, 24% had relief of SIBO but still had delayed OCTT, and three subjects (14%) had persistence of SIBO despite treatment with rifaximin.

Another condition that may predispose to SIBO by altering GI motility is hypothyroidism [2, 19, 76]. Several reports suggest that hypothyroidism reduces GI motility [92–94]. A study from Italy evaluated 50 patients with hypothyroidism and 40 controls for SIBO using GHBT [76]. Prevalence of SIBO was significantly higher in patients than in controls (54% vs. 5%). Abdominal discomfort, flatulence, and bloating were more frequent in patients who were positive for SIBO. These abdominal symptoms improved significantly after antibiotic therapy.

Some neurological conditions like Parkinson’s disease have also been reported to be associated with SIBO [2, 19, 29, 95, 96]. A study from Italy [97] evaluated 33 patients with Parkinson’s disease and 30 controls for SIBO. The prevalence of SIBO was significantly higher in patients than in controls (54.5% vs. 20.0%) and SIBO was associated with unpredictable motor fluctuations in patients with Parkinson’s disease. Moreover, the eradication of SIBO resulted in an improvement in motor fluctuations. A multicenter study evaluated the prevalence of SIBO in 103 patients with Parkinson’s disease using LHBT [98]. Prevalence of SIBO in Parkinson’s disease was 25.3% and SIBO independently predicted worse motor function. A recent case-control study from China evaluated 182 patients with Parkinson’s disease and 200 controls for SIBO using GHBT [99]. Prevalence of SIBO was significantly higher in patients with Parkinson’s disease than in controls (30.2% vs. 9.5%), and SIBO was associated with worse GI symptoms and worse motor function.

**Statement 9:** Drugs that retard GI motility, reduce acid, or break mucosal integrity may predispose to SIBO.

**Voting summary:** Accepted completely 68.4%, accepted with some reservation 31.6%.

**Level of evidence:** II-2.

**Grade of recommendation**: B.

Several classes of drugs may predispose to SIBO. These include drugs that slow down the gut motility like opioids, anticholinergics, antidiarrheals, and tricyclic antidepressants [2, 19, 21, 100, 101]. A study from Mayo Clinic (USA) evaluated clinical predictors of SIBO in duodenal aspirate culture and found narcotic use to be one of the factors associated with SIBO with odds ratio (OR) of 2.7 [35]. A recent study from the USA reported opioid use to be one of the predictors of SIBO in patients with chronic pancreatitis [77].

Drugs that break mucosal integrity of the small intestine may predispose to SIBO. These include primarily non-steroidal anti-inflammatory drugs (NSAIDs). Small intestinal injury induced by NSAIDs is dependent on bile secretion and is reported to augment the growth of several bacterial species, especially enterococci. An experimental rat study from Texas (USA) evaluated the relationship between indomethacin-related intestinal injury and SIBO [102]. The colony counts of ileal enterococci were significantly increased (500- to 1000-folds) in indomethacin-treated rats. Gut injury in these rats was associated with enterococcal overgrowth. Indomethacin-induced gut injury and bacterial overgrowth were independent of the route of administration of indomethacin. A study from Japan evaluated 43 patients taking NSAIDs for over 3 months [103]. All the subjects were examined with LHBT and video capsule endoscopy. Twenty-two (51%) patients had severe small intestinal damage. SIBO was detected in 5 of 21 patients (24%) without severe small intestinal damage and in 13 of 21 patients (59%) with severe small intestinal damage. SIBO on LHBT was significantly associated with an increased OR for severe small intestinal damage (OR, 6.54).

PPIs represent the prototype of acid-lowering drugs that have been shown in several studies to be associated with increased risk of SIBO [25, 60–64]. A few studies, however, have not supported this association [65–67]. PPIs are commonly prescribed drugs for several upper GI disorders like gastroesophageal reflux disease, *H. pylori–*related gastritis, gastroduodenal ulcers or erosions, and functional dyspepsia. PPIs are potent inhibitors of gastric acid secretion and since gastric acidity is an important gatekeeper defense against bacterial colonization in the upper GI tract, the use of PPIs, especially if use is prolonged [61], may increase the risk of bacterial overgrowth in the upper GI tract. Association of PPI use with SIBO has been shown by both breath testing [61, 62] and culture methods [25, 60, 63, 64].

An interesting study from India evaluated the impact of the addition of prokinetics to PPI on the frequency of SIBO. The authors observed that the group receiving PPI alone had a SIBO prevalence of 13.2% as against a prevalence of only 1.8% (*p*<0.05) in the group receiving PPI with prokinetics. The latter group also had faster OCTT as expected, and this was the possible reason for lower rates of SIBO in the dual therapy group [104]. A recent study utilizing a molecular technique also found increased bacterial load in PPI users as compared to non-PPI users, irrespective of comorbidities [4].

A meta-analysis of 11 studies showed an association of SIBO with PPI use when the upper gut aspirate culture was used for the diagnosis of SIBO [105]. A recent meta-analysis that included 19 studies and 7055 subjects also supported this association, but the pooled OR for this association was only 1.71, which is quite modest [106]. Subgroup analyses showed an association between SIBO and PPI use in studies that used aspirate culture and GHBT for diagnostic evaluation. A recent meta-analysis evaluated the prevalence and predictors of SIBO in IBS [36] and reported that PPI use was not associated with SIBO in patients with IBS.

**Statement 10:** A proportion of patients with functional GI disorders (FGIDs) especially those with IBS have been reported to be having SIBO.

**Voting summary:** Accepted completely 73.6%, accepted with some reservation 21.1%, accepted with major reservation 5.3%.

**Level of evidence:** II-2.

**Grade of recommendation**: A.

Several studies both in adults and children have reported SIBO in a fraction of patients with FGIDs, currently called disorders of gut-brain interaction, prominent among which by all means is IBS. In a study from Chile [107] in 367 patients with FGIDs, LHBT was used to evaluate for SIBO. SIBO was reported in 76% of subjects with IBS, 73% of those with functional constipation, 69% of those with functional diarrhea, and 68% of patients with functional bloating. In a study from the Netherlands [108] in pediatric patients with abdominal pain, GHBT was used for the evaluation of SIBO. SIBO was found in 14.3% of the subjects. IBS, altered defecation pattern, loss of appetite, and belching were predictors of the occurrence of SIBO. In a study from the USA, 75 children with chronic abdominal pain and 40 healthy controls were evaluated for SIBO using LHBT. The prevalence of abnormal LHBT in patients and controls was 91% and 35%, respectively [109]. In a study from India on 62 children with functional abdominal pain, SIBO as defined by abnormal GHBT was found in 17% [110].

A study from the USA evaluated 52 subjects with chronic functional bloating using a wireless motility capsule and LHBT. SIBO as defined by abnormal LHBT was found in 40% of cases and delayed GI motility was evidenced in 54% of cases [111]. In a recent study on functional bloating from Korea, SIBO was detected in 42.8% of patients, and SIBO was associated with significant dysbiosis as detected on fecal microbiota composition analysis by 16S ribosomal RNA amplification and sequencing [112]. A study from the USA evaluated 139 patients with unexplained gas, bloating, and diarrhea with duodenal aspirate culture and GHBT for the presence of SIBO. GHBT was positive in 27.3% of patients while culture was positive in 44.6% of subjects [16].

A case-control study from Brazil [113] evaluated 23 patients with functional dyspepsia and 11 controls for the presence of SIBO using LHBT. SIBO as defined by positive LHBT was observed in 56.5% of functional dyspepsia as compared to 0% in controls. The frequency of SIBO in functional dyspepsia subjects taking PPI was 75%. A study from Japan [114] evaluated the prevalence of SIBO by GHBT in patients with refractory FGIDs. Of the 38 FGID patients enrolled, 11 had functional dyspepsia, 10 had IBS, and 17 had IBS- functional dyspepsia overlap. SIBO was detected in overall 5.3% of the patients.

The FGID, which has attracted maximum global attention during the last two decades in terms of association with SIBO, is undoubtedly IBS. There are several seminal publications and exciting high-quality research in this particular arena, so much so that several meta-analyses are now available on this issue. Two most recent meta-analyses in the year 2020 highlighted some very interesting messages. The meta-analysis by Shah et al. [52] emphasized extensive variability in the prevalence of SIBO in IBS depending on the type of diagnostic technique used. Breath testing gave a yield of 35.5%, while culture gave a prevalence of 13.9% and 33.5% depending upon the use of a cut-off threshold of 10^5^ CFU/mL or 10^3^ CFU/mL, respectively. The meta-analysis by Ghoshal et al. [53] reported an overall prevalence of SIBO in IBS as 36.7%. Subjects with IBS were 2.6 and 8.3 times more likely to have SIBO as compared with healthy subjects using GHBT and upper gut aspirate culture, respectively. Subjects with IBS-D were more likely to have SIBO as per this meta-analysis. Interestingly, a recent study comparing the effect of antimicrobial therapy in patients with functional dyspepsia with and without concomitant IBS revealed that the improvement of dyspeptic symptoms was unrelated to the concomitant IBS symptoms suggesting that SIBO indeed is the cause of at least a subgroup of functional dyspepsia patients [115].

**Statement 11:** Fat in the small bowel induces ileal brake, which potentially promotes SIBO that may further lead to fat malabsorption.

**Voting summary:** Accepted completely 100%.

**Level of evidence:** II-2.

**Grade of recommendation**: B.

One of the postulated mechanisms of SIBO in patients with MAS (like tropical sprue, and celiac disease) is that intra-luminal unabsorbed fat induces slowing of proximal gut motility (also called ileal brake) through the liberation of hormones like peptide YY, neurotensin, and glucagon-like peptide [27, 78]. This ileal brake promotes SIBO, which may further promote fat malabsorption, resulting in a vicious cycle [27].

A landmark study from the UK [116] evaluated the possibility that malabsorbed fat in the ileum exerts an inhibitory feedback effect on intestinal motility. The investigators enrolled 24 healthy subjects and perfused the ileum with a fat-containing solution intended to produce ileal luminal fat levels similar to those in steatorrhea. Mean intestinal transit times through a saline-perfused jejunal segment were measured. After perfusion of fat into the ileum, mean transit times increased considerably. In addition, ileal fat perfusion resulted in a remarkable reduction of jejunal pressure wave activity. Ileal fat perfusion was also associated with a notable increase in plasma levels of neurotensin and enteroglucagon.

Another elegant study from the UK [117] evaluated the ileal brake effect of ileal infusion of partial digests of fat (oleic acid, triolein, medium-chain triglycerides). Marked reduction of jejunal pressure wave activity was observed after all three different lipid infusions. All three lipid infusions increased plasma levels of peptide YY (PYY), enteroglucagon, and neurotensin emphasizing the likely mechanism of the intestinal brake.

In the most comprehensive study to date on the issue of fat-induced ileal brake, Ghoshal et al. from India evaluated the effect of infusion of fat or placebo in the duodenum in patients with tropical sprue and healthy subjects on antroduodenal manometry, duodenocecal transit time (DCTT), and mediators of the ileal brake [28]. After fat infusion, proximal gut motility index was decreased as compared to fasting state in patients with tropical sprue, and DCTT was longer in patients as compared to healthy subjects. Fat infusion resulted in higher neurotensin and PYY levels in patients as compared to those in controls; and these levels were higher in patients with than those without SIBO.

**Statement 12:** Several systemic disorders (hepatic, pancreatic, others) are associated with SIBO.

**Voting summary:** Accepted completely 73.6%, accepted with some reservation 15.8%, accepted with major reservation 10.6%.

**Level of evidence:** II-2.

**Grade of recommendation**: A.

Though recent literature suggests an association of SIBO with several systemic disorders in terms of higher prevalence than in controls, this relationship with many of these systemic disorders is either multifactorial or unclear in terms of pathophysiology [1, 118]. Prominent among these disorders are hepatic diseases (cirrhosis, portal hypertension, NAFLD, and other CLDs), pancreatic disorders (acute and chronic pancreatitis), neurogenerative disorders (Parkinsonism, multiple sclerosis), cardiovascular disorders (coronary artery disease, and other atherosclerosis, deep venous thrombosis), end-stage renal disease, and other disorders like scleroderma, amyloidosis, human immunodeficiency virus infection, hypothyroidism, and diabetes mellitus [2, 19, 29, 39, 118]. Some of these associations are so well reported that even meta-analyses have been published regarding the relation of these disorders with SIBO.

SIBO has been frequently reported to be associated with CLD. Altered gut barrier function, reduced immunity, and impaired motility may contribute to the pathogenesis of SIBO in patients with CLD, especially those with cirrhosis and portal hypertension [39]. A recent meta-analysis by Shah et al. included 19 studies: 12 on cirrhosis, 5 on NAFLD/NASH, and 2 on CLD [49]. They reported a SIBO prevalence of 35.8% in studies that used breath tests, but the prevalence was as high as 68.3% in studies that used culture techniques. Pooled OR for the occurrence of SIBO was 7.15 as compared to controls. There was no significant difference between pooled prevalence in those with cirrhotic vs. non-cirrhotic CLD. Another meta-analysis from Russia [119] included 21 studies that involved 1264 patients with cirrhosis and 36 controls. The overall prevalence of SIBO in patients with cirrhosis and controls was 40.8% and 10.7%, respectively. Prevalence was 50.5% in those with decompensated cirrhosis. Pooled OR for the occurrence of SIBO was 6.83 as compared to controls. Predictors of SIBO in cirrhosis included the presence of minimal hepatic encephalopathy (MHE), ascites, spontaneous bacterial peritonitis (SBP), increased OCTT, and bacterial translocation. In a recent meta-analysis on the issue of SIBO in patients with NAFLD, Wijarnpreecha et al. evaluated 10 studies that included 1093 participants [120]. Significant association was found between NAFLD and SIBO with a pooled OR of 3.82.

Several studies have reported an increased frequency of SIBO in patients with chronic pancreatitis as compared to controls. The factors that may contribute to the pathogenesis of SIBO in these patients include impaired intestinal motility due to local inflammation, reduction in the secretion of pancreatic enzymes, effects of drugs like opioid analgesics, and in a few cases intestinal narrowing due to surrounding inflammatory process [39]. A meta-analysis from Italy evaluated 9 studies that included 336 patients [121]. They reported a pooled prevalence of SIBO as 36%. The pooled prevalence fell to 21.7% when studies using LHBT were excluded. Pooled OR for the occurrence of SIBO was 4.1. A recent meta-analysis from the USA included 13 studies with 518 patients with chronic pancreatitis [122]. They reported a pooled prevalence of SIBO as 38.6% with pooled OR of 5.58. A recent study by Chonchubhair el al. reported presence of diabetes, PPI use, alcoholic etiology, and use of pancreatic enzyme replacement therapy to be predictors of SIBO in patients with chronic pancreatitis [123]. One recent study from China reported SIBO to be associated with acute pancreatitis [124]. In this study, prevalence of SIBO in mild acute pancreatitis was lower (8.4%) than that in moderate (25.6%) or severe acute pancreatitis (25.9%). Also, frequency of SIBO was higher in those having organ dysfunction.

**Statement 13:** Lifestyle factors like chronic alcohol intake, and obesity may predispose to SIBO.

**Voting summary:** Accepted completely 31.6%, accepted with some reservation 52.6%, accepted with major reservation 15.8%.

**Level of evidence:** II-2.

**Grade of recommendation**: B.

Some lifestyle factors have been shown to increase the risk of occurrence of SIBO. One such factor is chronic alcohol intake. Chronic heavy alcohol intake has been reported to be associated with SIBO [125, 126]. This linkage can be explained by a variety of mechanisms as alcohol can modify intestinal defense through various mechanisms like direct toxic effect on the mucosal epithelium, decrease in the level of brush border enzymes, and induction of mucosal fibrosis [127–129]. Alcohol intake has also been shown to be associated with prolonged OCTT due to direct toxic damage of the intestinal smooth muscle [130]. Also, alcohol intake has been shown to adversely affect the intestinal mucosal immune system [131] predisposing to SIBO.

A study from Cleveland (USA) [132] evaluated the association between moderate alcohol consumption and SIBO. A total of 196 subjects were evaluated for SIBO using LHBT. Of them, 88 (45%) were alcohol consumers. Overall, the prevalence of positive LHBT was 47.4%. Among those who consumed alcohol, 58% had a positive LHBT, compared to 38.9% of abstainers (*p* = 0.008). There was a positive dose-response relationship between the amount of alcohol consumption and the positivity of LHBT [132].

With the background of the proposed metabolic role of gut microbiota in the pathogenesis of obesity [133, 134], several investigators tried to evaluate the relationship between obesity and SIBO. A few studies reported a positive relationship between these two entities [135–138] but one study from Korea noted an inverse relationship [139].

A recent meta-analysis evaluated the relationship between obesity and SIBO [140]. A total of five studies including 515 patients were included in this meta-analysis. The risk of SIBO among obese subjects was greater than in the non-obese but it did not reach statistical significance with a pooled OR of 2.08. When only Western studies were analyzed, the pooled OR became 3.41 and reached desired statistical significance.

**Statement 14:** Gut dysfunction in SIBO results from the altered luminal microenvironment, bacterial metabolites, altered motility and defense, and barrier dysfunction.

**Voting summary:** Accepted completely 63.1%, accepted with some reservation 21.1%, accepted with major reservation 15.8%.

**Level of evidence:** II-2.

**Grade of recommendation**: B.

Pathophysiology of gut dysfunction in SIBO is multifactorial [1, 29, 141]. Predominant mechanisms include gut inflammation, immune activation/dysfunction, altered motility, disturbed serotonergic activity, increased intestinal permeability, reduced luminal disaccharidase levels, deconjugation of bile salts, and nutrient and water malabsorption resulting in augmented intraluminal osmotic load [1, 142–145]. The metabolomic profile of intestinal contents in SIBO differs substantially from that in controls. An elegant study from India [146] did a H^1^-NMR spectroscopic analysis of the upper-gut aspirate in 31 patients with MAS with and without SIBO, and 10 control subjects. In comparison to control subjects, the patients with MAS had greater quantities of acetate, lactate, formate, and total bile acids/cholesterol in gut aspirate. Furthermore, MAS patients with SIBO had higher quantities of acetate, lactate, formate, and unconjugated bile acids than those without SIBO. In patients with MAS, the level of acetate correlated with the grade of SIBO, while the level of unconjugated bile salts correlated with the severity of steatorrhea, thereby indicating that bacteria in the small intestine generate acetate and induce deconjugation of bile salts, which in turn cause fat malabsorption resulting in steatorrhea.

Fat malabsorption in SIBO may also be associated with impaired absorption of lipid-soluble vitamins. The bacterial population in SIBO may induce vitamin B_12_ deficiency using ingested B_12_ to generate inactive cobamides, which in turn with dietary B_12_ intestinal binding sites, results in reduced B_12_ absorption and its deficiency [141, 147]. SIBO induces carbohydrate malabsorption by reducing enterocyte brush border disaccharidase activity [142]. Bacterial fermentation of carbohydrates into gas [145] results in bloating, distension, and abdominal discomfort. Protein malabsorption can result from multiple factors like decreased absorption of amino acids and peptides; decreased level of enterokinase, which may hamper activation of pancreatic proteases; and protein-losing enteropathy [148–150]. Increased intestinal permeability in SIBO patients has been shown to facilitate malabsorption and diarrhea [151]. This leaky gut in SIBO may contribute to mucosal immune activation resulting from the entry of luminal antigens into the mucosa [1, 152, 153]. Bacteria in SIBO may produce certain compounds that may have systemic effects. These agents include D-lactate, ammonia, ethanol, bacterial peptidoglycans, and endotoxins [29, 154]. These compounds are clinically relevant in the context of SIBO associated with short bowel syndrome [155]. A recent study from the USA described a syndrome of brain fogginess (BF) possibly related to SIBO and D-lactic acidosis in a cohort without short bowel syndrome patients [156].

**Statement 15:** Small Intestinal biopsy changes in patients with SIBO are non-specific.

**Voting summary:** Accepted completely 100%.

**Level of evidence:** II-2.

**Grade of recommendation**: C.

Morphologic changes associated with SIBO have not been studied extensively. Small intestinal histology in patients with SIBO may be either normal or may show subtle or non-specific abnormalities [141]. Since mucosal histology is quite variable, it is virtually impossible to diagnose SIBO conclusively on endoscopic biopsy [157].

An elegant experimental study using electron microscopy reported subtle enterocyte abnormalities like mitochondrial swelling and vacuolization of the microvillus membranes [158]. A recent experimental study in a post-infection IBS model of mice found a reduced density of interstitial cells of Cajal (ICC) and increased intra-epithelial lymphocyte (IEL) count in the ileum to be associated with the development of SIBO [159].

A study from Australia reported increased IEL and elevated IgA-containing plasma cells in the lamina propria as the standout features in the histology of small bowel in subjects with SIBO [160]. A study from the USA found that villous blunting was the only feature, which was more common in SIBO than in controls but more than half of biopsies from SIBO patients were histologically unremarkable [161].

**Statement 16:** Methane-producing bacteria slow gut transit and cause constipation.

**Voting summary:** Accepted completely 68.4%, accepted with some reservation 26.3%, accepted with major reservation 5.3%.

**Level of evidence:** II-2.

**Grade of recommendation**: A.

About 30% to 62% of healthy human beings have methane-producing bacteria in their gut [162]. Experimental and clinical studies indicate that methane inhibits GI motility and hence its concentration may inversely correlate with stool form and frequency [163, 164]. Also, the degree of breath methane production has been shown to correlate with the severity of constipation [163]. Moreover, therapy with antibiotics targeted to gut methanogens has been shown to improve intestinal transit as well as constipation [165]. A meta-analysis [166] established a significant association between methane on a breath test and constipation and also an association between methane and delayed transit. More recently, a systematic review and meta-analysis reported an association of methane positivity on breath testing with constipation-predominant IBS and inversely with IBD [167]. The North American Consensus defined a cut-off for high breath methane levels to be ≥10 parts per million (PPM) [18]. Excess methane production in the human gut is predominantly contributed by *Methanobrevibacter smithii*, which is a single-celled microorganism from the Archaea domain [168]. Since subjects with high breath methane may also have increased methanogen levels in stools, experts from North America have recently suggested a new terminology called “intestinal methanogen overgrowth (IMO)” [19].

There are limited studies on the role of methane in causing or promoting constipation. An experimental study from Korea [169] reported that infusion of methane significantly decreased peristaltic velocity and increased contraction amplitude of guinea pig ileum. Another interesting study from Korea [170] found breath methane positivity to be more frequent in patients with slow transit constipation than those with normal transit constipation and healthy subjects.

A landmark Indian study [171] found a greater number of *Methanobrevibacter smithii* in fecal samples of patients with IBS especially constipation-predominant IBS in comparison to healthy subjects. There was an inverse correlation between the copy number and the stool frequency, and the copy number was greater among methane producers than non-methane producers. The degree of breath methane correlated with the *M. smithii* copy number among methane producers. A recently published randomized controlled trial from India showed that reduction of breath methane using rifaximin shortens colonic transit time and improves constipation [172].

A recent meta-analysis on SIBO in IBS reported that methane-positive breath tests were more prevalent in IBS constipation than IBS diarrhea with an OR of 2.3 [52].

**Statement 17:** Small intestinal fungal overgrowth (SIFO) may coexist in patients with SIBO.

**Voting summary:** Accepted completely 57.9%, accepted with some reservation 26.2%, accepted with major reservation 5.3%, rejected with reservation 10.6%.

**Level of evidence:** II-2.

**Grade of recommendation**: B.

SIFO is an emerging entity. Very little published literature is available on this condition. Since risk factors for SIBO and SIFO are similar, both may coexist. Since normal intestinal propulsion helps to cleanse the intestine of bacteria and other microbes, intestinal stasis/dysmotility/post-surgical blind loop may predispose to SIBO as well as SIFO. In a study from the USA [63], GI dysmotility and PPI use were found to be independent predictors of SIBO or SIFO in patients with unexplained GI symptoms. Out of 150 evaluated patients, 94 (63%) had an overgrowth of whom 38/94 (40%) had SIBO, 24/94 (26%) had SIFO, and 32/94 (34%) had mixed SIFO/SIBO. Candida species were documented in those with SIFO. GI dysmotility was documented in 80/150 (53%) and PPI use was present in 65/150 (43%).

In another study from the USA [45], patients with unexplained abdominal pain, gas, bloating, and diarrhea, and without colectomy (controls), and with colectomy were evaluated for SIBO and SIFO. The severity of GI symptoms was greater in the post-colectomy group. Prevalence of SIBO (62% vs. 32%) SIFO/SIFO (24% vs. 8%) was also significantly higher in the post-colectomy group, indicating that colectomy predisposes to SIBO/SIFO and that these two may coexist.

### Clinical manifestations, differential diagnosis, and predictors

**Statement 18:** Clinical presentation may vary according to the severity of involvement and the underlying disease-causing SIBO.

**Voting summary:** Accepted completely 78.9%, accepted with some reservation 10.5%, accepted with major reservation 10.6%.

**Level of evidence:** II-2.

**Grade of recommendation**: B.

SIBO can present with a wide range of symptoms, many of which are non-specific and can occur in other GI disorders. Clinical symptoms may vary depending upon the severity of involvement and also the primary disease-causing bacterial overgrowth [1, 29]. The symptoms may occur due to malabsorption of nutrients, altered gut permeability, and effects of gut inflammation and immune activation. Subjects with SIBO may also be asymptomatic as illustrated in several case-control studies that have enrolled apparently healthy subjects as controls [29, 30].

Abdominal pain, bloating, gas, distension, flatulence, and diarrhea are the most common symptoms [16, 19, 63]. But no particular symptom can be specifically ascribed to SIBO. Symptoms often overlap with those of FGIDs. In severe cases, there may be additional clinical features like steatorrhea, peripheral edema, anemia, weight loss, deficiencies of fat- or water-soluble vitamins, especially vitamin D and B_12_, other micronutrient deficiencies, and failure to thrive in the pediatric population [1]. In addition to these symptoms, there may be apparent clinical features of the underlying disease predisposing to SIBO, like scleroderma, hypothyroidism, IBD, chronic pancreatitis, celiac disease, cirrhosis, and Parkinsonism [1, 19].

There may be additional systemic symptoms like fatigue, bodyache, poor concentration, and neurological symptoms [1, 19, 29]. Recently, a syndrome of BF, gas, and bloating was proposed as one of the presentations of SIBO [156, 173, 174]. D-lactic acidosis was more prevalent in BF compared to the non-BF group. None of these patients had short bowel, which is classically the situation predisposing to lactic acidosis and BF-like symptoms [29]. One recent study also suggested an association of SIBO with hyperammonemia encephalopathy [175].

**Statement 19:** Symptomatic patients with SIBO do not necessarily have nutrient deficiencies.

**Voting summary:** Accepted completely 84.2%, accepted with some reservation 15.8%.

**Level of evidence:** II-2.

**Grade of recommendation**: B.

Patients with SIBO generally have only mild non-specific symptoms like bloating, flatulence, abdominal discomfort, or distension with no sign of any nutrient deficit [16, 63]. This is the most usual clinical scenario of SIBO wherein the symptom profile mimics that of common FGIDs like IBS [21]. In contrast to this milder clinical profile, features of nutrient malabsorption and deficiencies like steatorrhea, anemia, sarcopenia, edema of lower extremities, neuropathy, metabolic bone disease, or tetany may be seen in more severe cases like the ones associated with scleroderma, jejuno-colonic fistulae, CIPO, intestinal strictures, or postsurgical blind loops [10, 19, 29, 71, 176].

The symptoms related to nutritional consequences of intestinal malabsorption occur over a substantial period of time and may result in significant malnutrition, weight loss, and growth failure (in children) [1]. Anemia in SIBO can be multifactorial. Iron, as well as vitamin B_12_, may be deficient due to malabsorption as well as sub-optimal intake due to associated anorexia or nausea [177]. Since iron deficiency causes microcytic anemia while B_12_ deficiency results in macrocytic anemia, the peripheral smear in SIBO subjects may show a dimorphic picture.

**Statement 20:** Physical examination may reveal features of predisposing conditions as well as sequelae of SIBO but it may also be entirely normal.

**Voting summary:** Accepted completely 84.2%, accepted with some reservation 15.8%

**Level of evidence:** II-2.

**Grade of recommendation**: B.

Physical examination findings in SIBO are non-specific [29]. It may be entirely normal in milder cases. The presence or absence of physical signs depends not only on the severity of SIBO but also on the type of primary underlying disease. The genesis of the physical signs may be related to malabsorbed nutrients, nutritional consequences of malabsorption, changes in gut permeability, systemic effects of intestinal inflammation, and immune activation resulting from pathologic bacterial fermentation in the small intestine [1, 19].

In severe cases, there may be features of malnutrition or malabsorption like anemia, pedal edema, sarcopenia, growth failure (in children), features of fat-soluble vitamin deficiency, tetany, polyneuropathy/other features of vitamin B_12_ deficiency, and features of other micronutrient deficiency [1]. Such florid signs are usually seen with certain particular causes of SIBO like a post-surgical blind loop, entero-colonic fistulae, or scleroderma [19, 71]. In addition to these features, the physical examination may reveal features of the associated/underlying disorders like hypothyroidism, CLD, Parkinsonism, scleroderma, rosacea, or scars of previous abdominal surgery if any.

**Statement 21:** Differential diagnosis may range from functional GI disorders in those with a milder clinical presentation to malabsorption syndrome in severe cases.

**Voting summary:** Accepted completely 94.7%, accepted with some reservation 5.3%.

**Level of evidence:** III.

**Grade of recommendation**: B.

The spectrum of differential diagnosis of SIBO is quite extensive since its clinical profile varies widely from asymptomatic or minimally symptomatic to full-blown features of malabsorption and malnutrition [19, 29]. The milder clinical version consists of non-specific symptoms like bloating, flatulence, distension, altered bowel habits, and abdominal discomfort or pain, which may mimic common FGIDs like IBS, dyspepsia, and functional bloating [16, 63]. The other extreme of the clinical scenario is epitomized by various features of MAS as can be seen with other causes of MAS like tropical sprue, celiac disease, hypogammaglobinemia, human immunodeficiency virus (HIV) infection, giardiasis, and strongyloidiasis [1, 19, 27, 178, 179]. In a study from India on 50 patients with MAS, SIBO was present in 42% cases [26]. Another study from the same center evaluated 13 patients with tropical sprue and found SIBO in 30.8% of these subjects [27]. A recent meta-analysis reported an overall pooled SIBO prevalence of 20% in patients with celiac disease which is an important cause of malabsorption all across the globe [180].

The features of malabsorption in SIBO may include steatorrhea, anemia, hypoproteinemia, sarcopenia, peripheral edema, weight loss or failure to thrive, and features of water- and fat-soluble vitamins especially vitamin B_12_ and vitamin D [1, 19, 29]. The patients with severe SIBO are usually associated with predisposing anatomic abnormalities like entero-colonic fistula, post-surgical blind loop, intestinal strictures, and small bowel diverticulosis; or major gut motility dysfunction like scleroderma or CIPO [71, 141]. Hence, SIBO must be considered in the differential diagnosis of any patient with malabsorption associated with a predisposing structural or functional cause. Recently, a few authors have suggested SIBO to be a possible etiopathogenetic link between post-infection IBS and post-infectious malabsorption syndrome (PI-MAS), traditionally known as tropical sprue [181].

**Statement 22:** Clinical predictors of SIBO in patients with IBS include female gender, old age, marked bloating and flatulence, long-term treatment with PPI, narcotic intake, low hemoglobin, and diarrheal subtype of IBS.

**Voting summary:** Accepted completely 68.4%, accepted with some reservation 26.3%, accepted with major reservation 5.3%.

**Level of evidence:** II-2.

**Grade of recommendation**: B.

SIBO in IBS is one of the most sought-after themes among FGID researchers during the last two decades [11, 19]. There are several publications including meta-analysis to suggest an association between these two entities; however, the puzzle is yet to be fully solved and several key aspects of this association need to be explored further [36, 51–53]. One key area of interest has been to explore the clinical predictors of SIBO in patients with IBS. The potential predictors include female gender, elderly age, long-term therapy with PPIs, narcotic intake, low hemoglobin, the diarrheal subtype of IBS, and presence of marked bloating or flatulence [1].

In a study from the USA, Reddymassu et al. evaluated 98 subjects with IBS for SIBO using GHBT [34]. A positive GHBT result was found to be more likely in patients with age >55 years and females (OR 4.0). Predominant bowel patterns or concurrent use of tegaserod, PPIs, or opiate analgesics were not found to be predictors of SIBO in this study.

In a study from India, Ghoshal et al. used GHBT to study SIBO in patients with IBS, those with chronic non-specific diarrhea (CNSD), and healthy controls (HC) [38]. Prevalence of SIBO in CNSD, IBS, and HC was 21.9%, 8.5%, and 2%, respectively. Older age and low hemoglobin were found to be predictors of SIBO in these patients.

A study from Mayo Clinic (USA) evaluated clinical predictors of SIBO by duodenal aspirate culture in the study population, which included IBS as well as non-IBS subjects [35]. Older age, narcotic use, and steatorrhea were associated with SIBO. Use of PPI was associated with bacterial overgrowth just falling short of meeting the criteria for SIBO.

In another study from India, Sachdeva et al. used GHBT to evaluate SIBO in patients with IBS and healthy controls [182]. SIBO was found to be more frequent in patients with IBS than HC (23.7% vs. 2.7%). Female gender, bloating, and IBS-D subtype were found to be predictors of SIBO in patients with IBS.

In one of the comprehensive studies published concerning SIBO in IBS, Ghoshal et al. used GHBT and LHBT as well as upper gut culture to evaluate SIBO in 80 patients with IBS [13]. A total of 15/80 (19%) patients were found to be having SIBO (bacterial counts ≥10^5^ CFU/mL on culture). SIBO was more common in IBS-D than in other IBS subtypes.

In a vital meta-analysis, Chen et al. analyzed 50 studies that have evaluated SIBO in IBS [36]. Overall pooled prevalence of SIBO in IBS was 38%, which was significantly higher than in healthy controls. Among patients with IBS, older age, female gender, and IBS-D subtype, but not PPI use, were associated with SIBO.

In a recent meta-analysis, Shah et al. evaluated 25 case-control studies with 3192 patients with IBS and 3320 controls [52]. Prevalence of SIBO in patients with IBS was 35.5% using hydrogen breath tests, 33.5% with culture using ≥10^3^ CFU/mL as cut-off, and 13.9% with culture cut-off of ≥10^5^ CFU/mL. OR for SIBO in IBS-D compared with IBS-C was 1.86. Use of PPI was not found to be associated with SIBO in patients with IBS (OR = 0.8).

In the most recent analysis, Ghoshal et al. evaluated 47 studies that have evaluated SIBO in IBS [53]. Overall pooled prevalence of SIBO in IBS was 36.7%. Patients with IBS-D were more likely to have SIBO as compared to other IBS subtypes.

### Investigations and treatment

**Statement 23:** The currently used tests for SIBO include GHBT and LHBT, and quantitative culture of upper gut aspirate.

**Voting summary:** Accepted completely 89.5%, accepted with some reservation 10.5%

**Level of evidence:** II-2.

**Grade of recommendation**: A.

Though quantitative upper gut aspirate culture is the current gold standard for the diagnosis of SIBO despite its limitation because only 30% of the gut bacteria can be cultured, hydrogen breath tests are popular due to their non-invasiveness [1]. Quantitative upper gut aspirate culture is better performed with a double-lumen catheter assembly that prevents contamination with nasopharyngeal flora (Ghoshal Gut Microbiota Sampler [GMS™]; patent application no. 20171104037). A cut-off of ≥10^5^ colony-forming unit (CFU)/mL is conventionally considered as diagnostic of SIBO [1, 183]. However, recently, even a lower level of bacterial overgrowth in the upper gut (≥10^3^ CFU/mL) has been found clinically important and hence, termed as low-grade SIBO [17]. In a North American consensus, a colony count ≥10^3^ CFU/mL has been considered diagnostic of SIBO. Even though only 30% of gut bacteria are cultured, the quantitative upper gut aspirate is considered the gold standard for diagnosis of SIBO. However, considering its invasiveness and need for considerable microbiological support, hydrogen breath tests are more popular in clinical practice. Recently, capsule measuring intraluminal hydrogen gas is being evaluated for diagnosis of SIBO and is being shown to be superior to breath hydrogen measurement [184].

**Statement 24:** Among non-invasive tests, GHBT is preferred over LHBT.

**Voting summary:** Accepted completely 78.9%, accepted with some reservation 21.1%.

**Level of evidence:** II-2.

**Grade of recommendation**: A.

The literature on this statement is reviewed along with that on statement 25.

**Statement 25:** Better tests for SIBO are needed as the culture of gut aspirate is invasive and more than 70% of microbes are non-culturable and the hydrogen breath tests are either insensitive or non-specific.

**Voting summary:** Accepted completely 89.4%, accepted with some reservation 5.3%, rejected completely 5.3%.

**Level of evidence:** II-1.

**Grade of recommendation**: A.

**Fig. 1:**
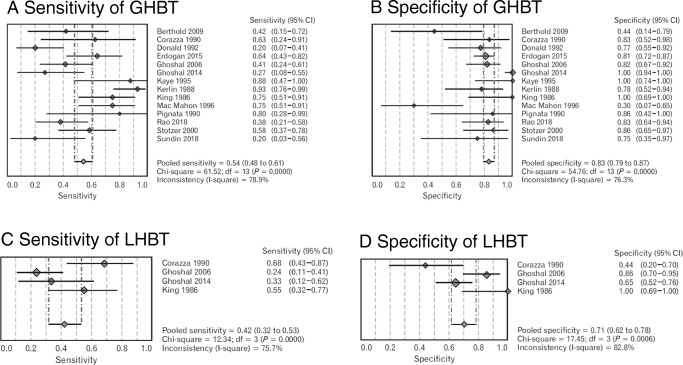
Forest plots of pooled sensitivity (**A**) and specificity (**B**) of glucose hydrogen breath test (GHBT); and pooled sensitivity (**C**) and specificity (**D**) of lactulose hydrogen breath test (LHBT). Reproduced from Losurdo et. al. [186]. It is an open-access article distributed under the terms of the Creative Commons Attribution Non-Commercial License (http://creativecommons.org/licenses/by-nc/4.0), which permits unrestricted non-commercial use, distribution, and reproduction in any medium, provided the original work is properly cited

Two commonly performed hydrogen breath tests include GHBT and LHBT. In GHBT and LHBT, fasting breath hydrogen and methane are estimated 3–4 times using a commercially available gas chromatograph [185]. In many machines, the value of carbon-di-oxide is also recorded, which is used as a correction factor for the adequacy of breath collection. Subsequently, the subject ingests either 75–100 g glucose dissolved in 150–200 mL water or 10 g lactulose contained in a 15-mL solution. End-expiratory breath hydrogen/methane is estimated every 10–15 min for 2–4 h. A rise in breath hydrogen 12 PPM above the average basal value following glucose ingestion is considered diagnostic of SIBO. The conventional criterion for diagnosis of SIBO on LHBT includes a double peak in breath hydrogen, the earlier one produced by the overgrown bacteria in the small bowel and the later one from the colon. Of late, an early-peak criterion for the diagnosis of SIBO has been proposed; it suggests that if a peak in breath hydrogen occurs within 90 minutes after lactulose ingestion, it is diagnostic of SIBO. However, this is based on the presumption that mouth-to-cecum transit time is always more than 90 minutes although several studies showed that mouth-to-cecum transit time may be much shorter [1, 48, 185]. Moreover, a few studies showed that when lactulose was co-administered with a radionuclide, in many subjects though peak the hydrogen occurred within 90 min after ingestion, the radionuclide was seen to have reached cecum. Accordingly, the early peak criterion to diagnose SIBO is not acceptable. In a recent meta-analysis, the sensitivity and specificity of GHBT were higher than LHBT (Fig. 1) [186]. Hence, GHBT should be preferred over LHBT for making the diagnosis of SIBO non-invasively. However, it is important to note that though the specificity of GHBT may reach 80% to 100%, its sensitivity has been reported to be as low as 30% to 40%; hence, though a positive GHBT is quite confirmatory of the presence of SIBO, a negative test may not mean the absence of it.

**Statement 26:** The role of newer diagnostic methods like culturomics, metabolomics, D-xylose, and ^13^C-based breath tests needs further evaluation.

**Voting summary:** Accepted completely 78.9%, accepted with some reservation 15.8%, rejected completely 5.3%.

**Level of evidence:** III.

**Grade of recommendation**: B.

In view of the low sensitivity and specificity of hydrogen breath tests, the invasive nature of quantitative upper gut aspirate culture, and the inability to culture as high as 70% gut bacteria, a search for better tests for the diagnosis of SIBO continues. D-xylose, a pentose sugar, is absorbed predominantly in the upper gut. D-xylose does not produce hydrogen due to bacterial fermentation as this sugar does not reach the large bowel in healthy subjects. Overgrown bacteria in the proximal gut in SIBO patients produce hydrogen by fermentation of D-xylose that is estimated in the exhaled breath. However, data on D-xylose breath tests are uncommonly reported. In a study, 513 (55%) of 932 consecutive subjects had SIBO on D-xylose breath test [187]. This was a retrospective study, and no gold standard method was used to verify the diagnosis of SIBO. Because bacteria in patients with SIBO deconjugate bile acids, the ^13^C or ^14^C glycocholic acid breath test has also been used for the diagnosis of SIBO [188]. This test involves ingestion of the ^14^C or ^13^C glycocholic acids, which are bile acids, and detection of ^14^CO_2_ or ^13^CO_2_ in the breath, which are expected to be high in SIBO patients [188]. However, data on the clinical utility of ^13^C or ^14^C glycocholic acid breath tests are scanty. In an earlier study, the lactose-^13^C ureide breath test was compared with GHBT, considering jejunal aspirate culture as the gold standard [189]. It had a sensitivity of 66.7% and a specificity of 100%, which were higher than GHBT.

**Statement 27:** Besides definitive tests for establishing SIBO, other ancillary tests are needed: (a) to investigate the underlying cause and (b) to evaluate for the sequelae.

**Voting summary:** Accepted completely 84.2%, accepted with some reservation 15.8%.

**Level of evidence:** III.

**Grade of recommendation**: B.

Table 2 lists the causes of SIBO. It is important to diagnose the causes of SIBO during careful history taking, physical examination, and appropriate investigations, as some of these disorders are potentially treatable. Treatment of the primary disorders may help to eradicate SIBO and prevent its recurrence and hence, finding out the underlying causes and mechanisms may have therapeutic implications. For example, prokinetic treatment delayed recurrence of SIBO after successful treatment with antibiotics in a retrospective study on patients with IBS with SIBO [190]. In another study on 15 patients with HIV-associated autonomic neuropathy with SIBO, pyridostigmine treatment alone resulted in the eradication of SIBO in 87% of patients during 2-month follow-up [191].

**Table 2:** Causes of small intestinal bacterial overgrowth

Structural abnormalities	Motility disorders	Biochemical abnormalities	GI and systemic diseases
Post-operative adhesion	Chronic intestinal pseudo-obstruction	Hypochlorhydria (e.g. atrophic gastritis, proton pump inhibitors therapy)	Connective tissue diseases (e.g. scleroderma)
Small bowel diverticula	Drugs (e.g., opiates, anticholinergics, tricyclic antidepressants)	Biliary diseases and cholecystectomy	Diabetic autonomic neuropathy, hypothyroidism
Small bowel stricture and fistulas	Irritable bowel syndrome and other functional bowel disorders		Tropical sprue, celiac disease and other causes of malabsorption syndrome
Blind loop syndrome	HIV-associated autonomic neuropathy		Chronic pancreatitis
Incompetent ileocecal valve	Parkinsonism, amyloidosis		Common variable immunodeficiency
Inflammatory bowel disease, particularly Crohn disease			Cirrhosis of liver
			Non-alcoholic fatty liver disease
			Obesity and its surgical treatment

*GI* gastrointestinal, *HIV* human immunodeficiency virus

**Statement 28:** Key components of management of SIBO include the followings: (a) treatment of predisposing conditions, (b) appropriate antibiotics, and (c) correction of nutritional deficiencies.

**Voting summary:** Accepted completely 89.5%, accepted with some reservation 10.5%.

**Level of evidence:** I.

**Grade of recommendation**: A.

The literature on this statement is reviewed along with that on statement 29.

**Statement 29:** Though small bowel bacteria in patients with SIBO are sensitive in vitro to several antibiotics, rifaximin may be preferred due to its broad spectrum and lack of systemic adverse effects.

**Voting summary:** Accepted completely 78.9%, accepted with some reservation 10.5%, Accepted with major reservation 10.6%.

**Level of evidence:** I.

**Grade of recommendation**: A.

Treatment of primary disorders predisposing to the development of SIBO, if any (Table 2), not only helps to alleviate the condition but also prevents its recurrence following successful treatment. Hence, attention to detecting these predisposing conditions and their appropriate treatment is essential.

**Fig. 2:**
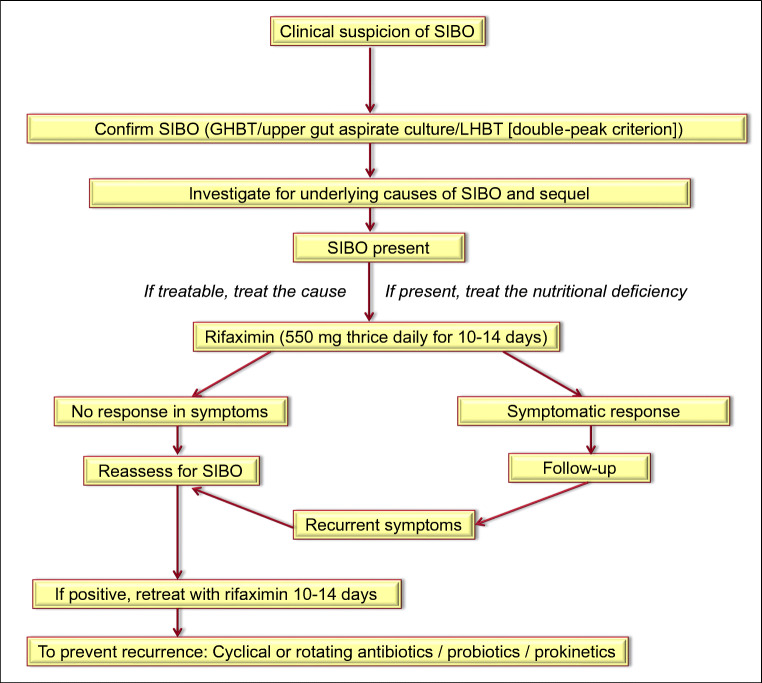
Outline of suggested management of a patient with suspected small intestinal bacterial overgrowth (SIBO). *GHBT* glucose hydrogen breath test; *LHBT* lactulose hydrogen breath test

Though several methods to manage the SIBO may be potentially useful, the broad-spectrum gut-specific antibiotic rifaximin is the most studied drug. Figure 2 presents a simplified algorithm for diagnosis and pharmacotherapy of SIBO. Several other antibiotics have also been tried in the treatment of SIBO such as norfloxacin, ciprofloxacin, tetracycline, doxycycline, neomycin, co-trimoxazole, ampicillin-clavulanic acids, and metronidazole. In a study from India, the bacterial populations causing SIBO were found to be more often sensitive to fluoroquinolones than to other antibiotics [26]. However, rifaximin was not evaluated in that study.

Rifaximin (C_43_H_51_N_3_O_11_), a structural analogue of rifampin that inhibits bacterial RNA synthesis by binding to the DNA-dependent RNA polymerase, is a non-absorbable antimicrobial drug effective against both aerobic and anaerobic Gram-positive as well as Gram-negative bacteria [1]. In a meta-analysis of 32 studies including 1331 patients with SIBO, frequency of eradication SIBO with rifaximin treatment by intention-to-treat analysis was 70.8% (95% CI: 61.4–78.2; I2 to 89.4%) and by per-protocol analysis 72.9% (95% CI: 65.5–79.8; I2 to 87.5%) [192]. In another meta-analysis of five non-randomized studies on patients with SSc including 78 patients with SIBO, though the antibiotics were found to eradicate SIBO, the probiotics and prokinetics were of uncertain efficacy [193].

IBS, a common disorder of gut-brain interaction, is known to be associated with SIBO [53]. The popularity of rifaximin among gastroenterologists and physicians was brought in by a RCT of this drug in the management of IBS that showed rifaximin to be 41% effective to relieve the symptoms of IBS in contrast to 31% efficacy of placebo that led to the approval of the drug in the treatment of non-constipated IBS patients by the Food and Drug Administration of USA [194]. However, that study did not evaluate for SIBO in IBS patients and hence, treatment response in relation to the presence and absence of SIBO was not known. In a study, it was found that the bacterial populations causing SIBO are more often responsive to fluoroquinolones than to other antibiotics [26].

A meta-analysis of eight studies showed that the overall normalization rate of breath test with rifaximin was 49.5% (95% CI, 44.0 to 55.1). Antibiotics like metronidazole, neomycin, and ciprofloxacin (four studies) showed a higher response rate than placebo in normalizing breath tests with an OR of 2.55 (95% CI, 1.29 to 5.04) [195].

Currently, most patients with SIBO may be diagnosed much before severe nutritional deficiencies develop. However, nutritional deficiencies, if present, must be identified and treated appropriately. Whereas vitamin B_12_ deficiency is common among these patients, serum folate levels may be normal or high due to the bioavailability of bacterially synthesized folic acid except in patients with tropical sprue who often have folate deficiency [179]. Vitamin B_12_ should be supplemented in patients with SIBO parenterally.

**Statement 30:** Patients with IBS with SIBO show a better response to antibiotics as compared to those without SIBO.

**Voting summary:** Accepted completely 100%.

**Level of evidence:** I

**Grade of recommendation**: A

In a proof of concept double-blind randomized-controlled trial, one of the authors found that SIBO was highly reliable to predict response to treatment with antibiotics among IBS patients [14]. In this study, 80 patients with IBS diagnosed by Rome III criteria were evaluated for SIBO (≥10^5^ CFU/mL) by upper gut aspirate culture; patients with and without SIBO were separately randomized (stratified randomization) either to norfloxacin 400 mg twice daily and placebo. 87.5% of 15 patients with SIBO responded to treatment at 1 month in contrast to 25% of 65 without SIBO. This study [14] evaluated norfloxacin instead of rifaximin as the latter drug was not available in India at the time of study. Two subsequent studies, one from China [196] and another from the USA [197], further reproduced similar observations. However, both these studies used hydrogen breath tests to diagnose SIBO. More studies are needed on this issue.

**Statement 31:** Patients with slow transit chronic constipation associated with high breath methane on LHBT may respond to treatment with rifaximin.

**Voting summary:** Accepted completely 57.9%, accepted with some reservation 31.6%, accepted with major reservation 10.6%.

**Level of evidence:** I.

**Grade of recommendation**: B.

Several studies and meta-analysis showed that excess breath methane on LHBT is associated with chronic constipation and slow colon transit [166]. Three RCT, two from the USA [165, 198] and one from India [172], showed that reduction in breath methane by treatment with antibiotics such as rifaximin, neomycin, or a combination of these two was associated with improvement of constipation. One study also showed that reduction in breath methane by rifaximin treatment was associated with acceleration of colon transit [172]. More studies are needed on this issue.

**Statement 32:** SIBO is known to recur following successful treatment with rifaximin.

**Voting summary:** Accepted completely 78.9%, accepted with some reservation 21.1%

**Level of evidence:** II-2.

**Grade of recommendation**: A.

The literature on this statement is reviewed along with that on statement 33.

**Statement 33:** Predictors of recurrence of SIBO include older age, long-term treatment with PPIs, prior abdominal surgery, and persistence of predisposing condition.

**Voting summary:** Accepted completely 68.4%, accepted with some reservation 31.6%.

**Level of evidence:** II-2.

**Grade of recommendation**: B.

SIBO often recurs following its successful treatment, particularly in absence of a cause or in presence of a treatable predisposing condition that has not been treated. In an Italian study, 12.6%, 27.5%, and 43.7% of 80 patients with SIBO successfully treated with rifaximin had a recurrence of it on GHBT at 3-, 6-, and 9-month follow-up, respectively [199]. Older age, history of appendectomy, and long-term PPI intake were associated with recurrence during follow-up [199]. Recurrence of SIBO was often associated with recurrence of GI symptoms. In an Indian study, of 78 patients with SIBO diagnosed by GHBT and successfully treated with rifaximin, 18% and 43% had recurrence at 3 and 6 months, respectively [200].

**Statement 34:** Drugs promoting GI motility and treating the predisposing conditions may prevent the recurrence of SIBO.

**Voting summary:** Accepted completely 31.6%, accepted with some reservation 52.6%, accepted with major reservation 15.8%.

**Level of evidence:** III.

**Grade of recommendation**: C.

Since SIBO recurrence after successful treatment is quite high (almost 40% during 6 months) [200], the prevention of recurrence is of utmost importance. Since small bowel stasis predisposes to SIBO, increasing gut motility should prevent SIBO recurrence. In a retrospective study, recurrence of symptoms presumed to result from SIBO was delayed among patients treated with tegaserod or erythromycin than those without these drugs [190]. In another study, 15 patients with well-controlled HIV infection with autonomic neuropathy and SIBO have treated with pyridostigmine 30 mg thrice daily for 2 months. GHBT, gastric emptying (GE), plasma sCD14 (a marker of macrophage activation and indirect measure of translocation), interleukin (IL)-6, tumor necrosis factor-alpha (TNFα), and GI autonomic symptoms were compared before and after treatment [191] The authors found that SIBO improved in 13 (87%) patients; though GE did not improve, (TNF-α) and sCD14 levels declined by 12% and 19% (*p*<0.05 for both), and IL-6 or GI symptoms were comparable. Though this study was done on a small sample of patients, it did support the possibility of beneficial effect of improving gut motility on patients with SIBO.

**Statement 35:** Patients with recurrent symptoms following response to rifaximin respond to re-treatment with it.

**Voting summary:** Accepted completely 57.9%, accepted with some reservation 36.8%, accepted with major reservation 5.3%.

**Level of evidence:** II-1.

**Grade of recommendation**: B.

The data on retreatment of SIBO after recurrence following successful treatment is limited. No study has yet been reported on the efficacy of rifaximin in the re-treatment of recurrent SIBO. Since an earlier study showed that recurrent SIBO after successful treatment with rifaximin was associated with recurrence of symptoms [199], the TARGET 3 study that evaluated the efficacy of rifaximin for recurrent symptoms after successful rifaximin treatment for IBS may be considered as the evidence for the above statement. TARGET 3 study showed the safety and efficacy of re-treatment with rifaximin among 636 patients with IBS-D, who had previously responded to rifaximin but developed recurrent IBS symptoms over 18 weeks follow-up [201]. Another study showed that short-term re-treatment of these patients with rifaximin did not alter the stool microbial susceptibility to rifaximin, rifampin, and non-rifamycin antibiotics [202]. More studies are needed on this issue.

**Statement 36:** Probiotics may be of some benefit but more evidence is required.

**Voting summary:** Accepted completely 100%.

**Level of evidence:** II-2.

**Grade of recommendation**: B.

Despite the ability of probiotics to alter gut microbiota, their efficacy in treatment of SIBO is not encouraging. In a recent meta-analysis [203], of the five studies on probiotics including 266 patients with SIBO diagnosed using hydrogen breath test (three studies used probiotics only, and two with antibiotics), two studies comparing probiotics and placebo showed a positive result and the other one showed a negative result. Both the studies on probiotics and antibiotics showed that probiotic treatment in addition to antibiotics was superior. In another randomized crossover trial not included in this meta-analysis on 10 SIBO patients treated with norfloxacin, amoxicillin-clavulanic acid, and *Saccharomyces boulardii* over 7 days, though both the antibiotics conferred a positive result, *S. boulardii* was not useful [204]. More studies are needed on this issue.

**Statement 37:** The role of the low fermentable oligosaccharides, disaccharides, monosaccharides and polyols (FODMAP) diet in the management of SIBO needs further evaluation.

**Voting summary:** Accepted completely 63.2%, accepted with some reservation 26.2%, accepted with major reservation 5.3%, rejected with reservation 5.3%.

**Level of evidence:** III.

**Grade of recommendation**: C.

Since dietary modification is known to alter gut microbiota [205, 206], changing the diet may be a potential method to treat SIBO. However, the data on this are scanty. A single study evaluated the role of an elemental diet in patients with SIBO. The authors concluded that an elemental diet was effective in normalizing an abnormal LHBT in patients with IBS, who also experienced symptomatic improvement [207]. More studies are needed on this issue.

## Declarations

### Copyright

Indian Neurogastroenterology and Motility Association.

### Disclaimer

The authors are solely responsible for the data and the contents of the paper. In no way, the Honorary Editor-in-Chief, Editorial Board Members, the Indian Society of Gastroenterology or the printer/publishers are responsible for the results/findings and content of this article.
